# Male Infertility Diagnosis: Improvement of Genetic Analysis Performance by the Introduction of Pre-Diagnostic Genes in a Next-Generation Sequencing Custom-Made Panel

**DOI:** 10.3389/fendo.2020.605237

**Published:** 2021-01-26

**Authors:** Vincenza Precone, Rossella Cannarella, Stefano Paolacci, Gian Maria Busetto, Tommaso Beccari, Liborio Stuppia, Gerolamo Tonini, Alessandra Zulian, Giuseppe Marceddu, Aldo E. Calogero, Matteo Bertelli

**Affiliations:** ^1^ MAGI EUREGIO, Bolzano, Italy; ^2^ Department of Clinical and Experimental Medicine, University of Catania, Catania, Italy; ^3^ MAGI’S LAB, Rovereto, Italy; ^4^ Department of Urology, “Sapienza” University of Rome, Policlinico Umberto I, Rome, Italy; ^5^ Department of Pharmaceutical Sciences, University of Perugia, Perugia, Italy; ^6^ Department of Psychological, Health and Territorial Sciences, School of Medicine and Health Sciences, “G. d’Annunzio” University of Chieti-Pescara, Chieti, Italy; ^7^ Department of Surgery, Fondazione Poliambulanza, Brescia, Italy; ^8^ EBTNA-LAB, Rovereto, Italy

**Keywords:** male infertility, next-generation sequencing, genetic test, spermatogenesis defects, azoospermia, oligozoospermia

## Abstract

**Background:**

Infertility affects about 7% of the general male population. The underlying cause of male infertility is undefined in about 50% of cases (idiopathic infertility). The number of genes involved in human spermatogenesis is over two thousand. Therefore, it is essential to analyze a large number of genes that may be involved in male infertility. This study aimed to test idiopathic male infertile patients negative for a validated panel of “diagnostic” genes, for a wide panel of genes that we have defined as “pre-diagnostic.”

**Methods:**

We developed a next-generation sequencing (NGS) gene panel including 65 pre-diagnostic genes that were used in 12 patients who were negative to a diagnostic genetic test for male infertility disorders, including primary spermatogenic failure and central hypogonadism, consisting of 110 genes.

**Results:**

After NGS sequencing, variants in pre-diagnostic genes were identified in 10/12 patients who were negative to a diagnostic test for primary spermatogenic failure (n = 9) or central hypogonadism (n = 1) due to mutations of single genes. Two pathogenic variants of *DNAH5* and *CFTR* genes and three uncertain significance variants of *DNAI1*, *DNAH11*, and *CCDC40* genes were found. Moreover, three variants with high impact were found in *AMELY*, *CATSPER 2*, and *ADCY10* genes.

**Conclusion:**

This study suggests that searching for pre-diagnostic genes may be of relevance to find the cause of infertility in patients with apparently idiopathic primary spermatogenic failure due to mutations of single genes and central hypogonadism.

## Introduction

The increasing knowledge of male reproduction physiology, of fertilization, and the advent of increasingly effective assisted reproductive techniques, have led to a profound change in the management of male infertility. Currently, the diagnostic workflow offered to male infertile patients includes medical history collection and physical examination, followed by a combination of laboratory testing tailored to each case, including an in-depth genetic laboratory analysis (1–3). Diagnostic tests should be performed after at least 1 year of infertility. Accordingly, a couple can be defined infertile if they do not reach pregnancy after a year of unprotected and regular sexual intercourses (4).

Genetic factors are found in about 15% of male infertile patients. They include chromosomal abnormalities or single-gene mutations (5, 6). Over 200 genetic disorders related to male infertility are reported in the Online Mendelian Inheritance in Man (OMIM) database (7, 8). The genetic of male infertility is greatly complex because semen and testis histological phenotypes are very heterogeneous and up to 2,300 genes are involved in spermatogenesis (1, 9). Moreover, studies in male infertility are challenging. Accordingly, genetic infertility results in an elimination of these mutations from the gene pool, since these are not transmitted. Furthermore, genetic and epigenetic changes accumulate in spermatozoa with aging, and rare single nucleotide polymorphisms and copy number variants can contribute to idiopathic male infertility (1). It is important to trace the non-genetic and genetic causes of male infertility since the latter are the cause of half of the cases of non-conception (4). Notably, to identify new genetic biomarkers of genetic infertility deserve investigation, because the standard clinical evaluation of infertile patients and karyotype analysis can identify the cause of infertility only in about 50% of the cases (10). The combination of genetic and epigenetic testing seems to identify genetic variations and differential expression of specific genes, providing information on the true ability of a man to reproduce. In contrast, a semen analysis may fail to evidence even a partial impairment of sperm parameters (9).

There are two general approaches for finding genes involved in infertility: the candidate gene approach in model animals, and the whole genome studies such as single-nucleotide polymorphism microarray and next-generation sequencing (NGS) technologies, such as exome or whole-genome sequencing (11, 12). Despite a throughout diagnostic workup, conventional genetic tests largely fail to reach a diagnosis (13) and the cause of male infertility remains elusive in up to ∼70% of cases (14). Recent research seems to address the role of NGS technology in raising the rate of diagnosis in male infertility (15, 16). Accordingly, several diagnostic genes have already been shown to be involved in the pathogenesis of male infertility (15). Pre-diagnostic genes, including those reported in association with male infertility but with no definitive evidence of a causative role, may help to reach a diagnosis. To this end, the present study was undertaken to evaluate a series of pre-diagnostic genes by comparing the results with those obtained with our usual NGS custom-made gene panel for the diagnosis of male infertility, including 110 genes.

## Methods

### Patients and Samples

Twelve patients with a clinical diagnosis of male infertility and negative to diagnostic genetic testing were selected for this study. Eleven were suspected to have primary spermatogenic failure and one was suspected to have central hypogonadism. More in detail, primary spermatogenic failure was suspected for a history of couple infertility longer than 2 years, after the exclusion of the female factor infertility and of acquired causes of male infertility (e.g. male accessory gland infection, varicocele, testicular trauma, etc.). Also, patients enrolled in this study were negative for first step genetic analysis, such as karyotype abnormalities, Y chromosome AZF microdeletions, or *CFTR* conventional gene mutations.

An informed written consent was obtained from each patient. The study was carried out following the tenets of the Declaration of Helsinki and it was approved by the local Ethics Committee. A blood EDTA sample was collected from each subject. Samples of genomic DNA of all subjects were extracted from peripheral blood using a commercial kit (SAMAG 120 BLOOD DNA Extraction Kit). DNA was quantified using Quant-iT Picogreen dsDNA Assay Kit (Life Sciences) and a Varioskan LUX (Thermo Scientific).

### Gene Panel Design

A single NGS panel related to male infertility disorders comprising a total of 175 genes was designed. Then, 110 genes were analyzed in a diagnostic setting, and 65 genes comprising pre-diagnostic or informative genes were analyzed in patients who resulted negative to the diagnostic testing. The genes included in the panel were based on their correlation with male infertility described in Online Mendelian Inheritance in Man (OMIM) (7), GeneReviews (17), and primary literature. Genes were classified as “diagnostic” when they and their genetic variants were clearly correlated to male infertility in literature. Instead, genes were classified as “informative or pre-diagnostic” when they were reported to be associated with male infertility, but the causality link has not been unequivocally established. The list of genes associated with male infertility related to the diagnostic suspect of the considered subjects included in the two NGS panel, is shown in **Table 1**.

**Table 1:** Diagnostic and pre-diagnostic genes associated with male infertility included in the custom NGS panels.

Diagnostic and pre-diagnostic genes (Male condition)	Genes (coverage)	OMIM	REFSEQ
**Diagnostic genes (Defects of primary spermatogenesis)**	*AURKC* *CATSPER1* *CFAP44* *DPY19L2* *KLHL10* *NANOS1* *PICK1* *PLK4* *SEPT12* *SOHLH1* *SUN5* *SYCP3* *TEX11* *USP9Y* *ZPBP* *BRDT* *CFAP43* *DNAH1* *HSF2* *MEIOB* *NR5A1* *PLCZ1* *RHOXF2* *SLC26A8* *SPATA16* *SYCE1* *TAF4B* *TEX15* *ZMYND15*	*603495 *606389 *617559 *613893 *608778 *608226 *605926 *605031 *611562 *610224 *613942 *604759 *300311 *400005 *608498 *602144 *617558 *603332 *140581 *617670 *184757 *608075 *300447 *608480 *609856 *611486 *601689 *605795 *614312	NM_001015878 NM_053054 NM_018338 NM_173812 NM_152467 NM_199461 NM_012407 NM_014264 NM_144605 NM_001012415 NM_080675 NM_001177948 NM_001003811 NM_004654 NM_007009 NM_001726 NM_025145 NM_015512 NM_004506 NM_152764 NM_004959 NM_033123 NM_032498 NM_052961 NM_031955 NM_130784 NM_005640 NM_001350162 NM_001136046
**diagnostic genes (Hypogonadotropic hypogonadism)**	*ANOS1*	*300836	NM_000216
	*CCDC141*	*616031	NM_173648
	*DUSP6*	*602748	NM_001946
	*FGF17*	*603725	NM_003867
	*(100.0%)*	*136350	NM_023110
	*FGFR1*	*136530	NM_000510
	*(100.0%)*	*138850	NM_000406
	*FSHB*	*606807	NM_017563
	*(100.0%)*	*604161	NM_032551
	*GNRHR*	*608137	NM_015537
	*(100.0%)*	*607002	NM_021935
	*IL17RD*	*603961	NM_006080
	*(100.0%)*	*610224	NM_001012415
	*KISS1R*	*607984	NM_030964
	*(84.84%)*	*603819	NM_001035235
	*NSMF*	*162332	NM_001059
	*(95.03%)*	*109135	NM_021913
	*PROK2*	*608892	NM_017780
	*(97.67%)*	*613301	NM_001024613
	*SEMA3A*	*600483	NM_033163
	*(100.0%)*	*604808	NM_198391
	*SOHLH1*	*152760	NM_001083111
	*(100.0%)*	*604846	NM_004807
	*SPRY4*	*603286	NM_002256
	*(98.25%)*	*152780	NM_000894
	*SRA1*	*607002	NM_001126128
	*(100.0%)*	*607123	NM_144773
	*TACR3*	*608166	NM_012431
	*(100.0%)*	*602229	NM_006941
	*AXL*	*607984	NM_001293290
	*(100.0%)*	*162330	NM_013251
	*CHD7*	*606417	NM_018117
	*(99.54%)* *FEZF1* *(96.46%)* *FGF8* *(93.16%)* *FLRT3* *(100.0%)* *GNRH1* *(100.0%)* *HS6ST1* *(96.3%)* *KISS1* *(100.0%)* *LHB* *(100.0%)* *PROK2* *(97.67%)* *PROKR2* *(100.0%)* *SEMA3E* *(100.0%)* *SOX10* *(100.0%)* *SPRY4* *(98.25%)* *TAC3* *(100.0%)* *WDR11* *(100.0%)*		
**Pre-diagnostic genes**	*ADGRG2* *CFTR* *NLRP14* *RBMXL2* *INHBB* *INSL6* *FKBPL* *KLK12* *KLK14* *KLK15* *KLK3* *KLK4* *KLK6* *SEMG1* *TSPY1* *PRM1* *PRM2* *NPAS2* *CFAP65* *DNAH6* *TDRD9* *RSPH1* *CCDC40* *CCDC39* *SPAG17* *DNAH10* *CCDC103* *GAS8* *DNAH5* *DNAI1* *AURKB* *CAMK4* *DPP6* *HORMAD1* *MAGEB4* *PIWIL1* *PYGO2* *SPINK2* *TNP1* *TSPYL1* *E2F1* *USP26* *FKBP6* *NR0B1* *WT1* *NSUN7* *DNAH11* *GALNTL5* *GAPDHS* *TEKT2* *ADCY10* *PLA2G6* *CATSPER2* *CATSPER4* *CATSPER3* *BSCL2* *NXF3* *PRMT7* *ANKS1A* *TSPAN7* *SPANXN5* *SSX7* *AMELY* *EPHA3* *H2BFWT*	*300572 *602421 *609665 *605444 *147390 *606414 *617076 *605539 *606135 *610601 *176820 *603767 *602652 *182140 *480100 *182880 *182890 *603347 *614270 *603336 *617963 *609314 *613799 *613798 *616554 *605884 *614677 *605178 *603335 *604366 *604970 *114080 *126141 *609824 *300153 *605571 *606903 *605753 *190231 *604714 *189971 *300309 *604839 *300473 *607102 *617185 *603339 *615133 *609169 *608953 *605205 *603604 *607249 *609121 *609120 *606158 *300316 *610087 *608994 *300096 *300668 *300542 *410000 *179611 *300507	NM_001079858 NM_000492 NM_176822 NM_014469 NM_002193 NM_007179 NM_022110 NM_019598 NM_022046 NM_017509 NM_145864 NM_004917 NM_002774 NM_003007 NM_003308 NM_002761 NM_001286356 NM_002518 NM_194302 NM_001370 NM_153046 NM_001286506 NM_001243342 NM_181426 NM_206996 NM_001372106 NM_213607 NM_001286205 NM_001369 NM_012144 NM_004217 NM_001744 NM_130797 NM_032132 NM_002367 NM_001190971 NM_138300 NM_021114 NM_003284 NM_003309 NM_005225 NM_031907 NM_003602 NM_000475 NM_000378 NM_024677 NM_003777 NM_145292 NM_014364 NM_014466 NM_018417 NM_001004426 NM_054020 NM_198137 NM_178019 NM_032667 NM_022052 NM_019023 NM_015245 NM_004615 NM_001009616 NM_173358 NM_001143 NM_005233 NM_001002916

**Figure 1:**
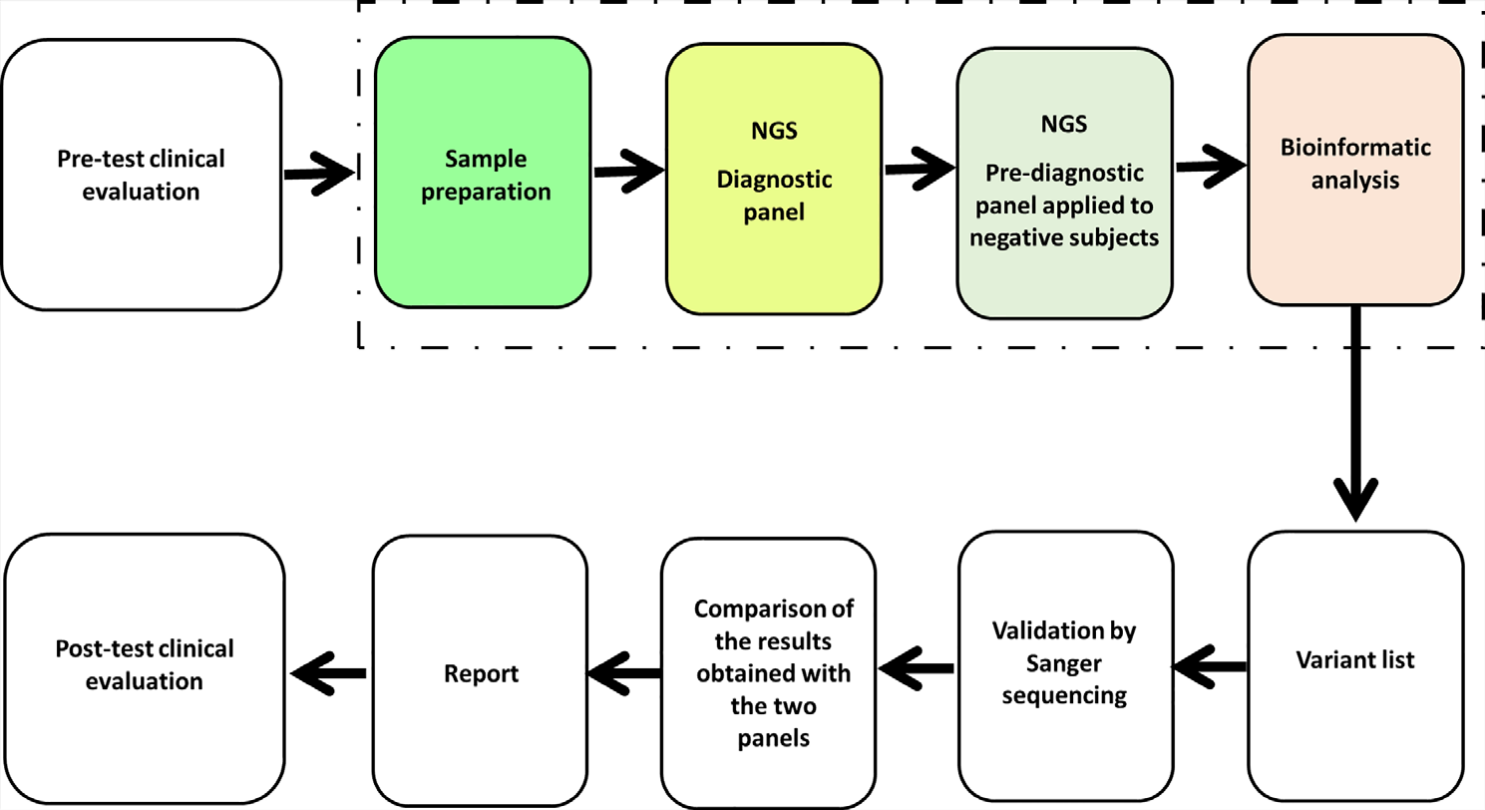
Laboratory and analysis workflow.

The custom Illumina Nextera panel included genomic targets comprising coding exons and 15 bp flanking regions of each gene. The target length of the diagnostic panel was 314,814 bp. Instead, the target length of the pre-diagnostic panel was 188,074 bp. **Figure 1** describes the laboratory and analysis workflow.

### Genetic Analysis and Variant Detection

DNA samples were processed using MiSeq personal sequencer (Illumina, San Diego, CA, USA) using a paired-end protocol and a 150 bp long reads, following the laboratory methods described elsewhere (18, 19). Fastq (forward-reverse) files were obtained after sequencing. Reads alignment was done by the BWA (0.7.17-r1188) software. Duplicates were removed using the SAMBAMBA (0.6.7) program and GATK (4.0.0.0) were used for re-alignment. We used international databases dbSNP (www.ncbi.nlm.nih.gov/SNP/) and Human Gene Mutation Database professional (HGMD; https://apps.ingenuity.com/ingsso/login) for all nucleotide changes. *In silico* evaluation of the pathogenicity of nucleotide changes in exons was performed using Polymorphism Phenotyping v2 (PolyPhen-2, http://genetics.bwh.harvard.edu/pph2/), Sorting Intolerant from Tolerant (SIFT, https://sift.bii.a-star.edu.sg/), and MutationTaster (http://www.mutationtaster.org). Minor allele frequencies (MAF) were checked in the Genome Aggregation Database gnomAD (http://gnomad.broadinstitute.org/). Sanger sequencing was performed for confirmation when target region coverage was less than 15 reads. Nucleotide alterations were analyzed and validated by Sanger sequencing. After confirmation, each variant was classified as a pathogenic, likely pathogenic, variant of unknown significance (VUS), likely benign, or benign, according to the American College of Medical Genetics (ACMG) guidelines (20). Coding genomic regions (CDS) that were sequenced with coverage less than 15X were eventually re-sequenced using Sanger technology.

## Results

Twelve infertile patients were analyzed with two NGS custom-made panels. They had a median age of 38 years (range 24–55). Clinical details, including testicular histology and responsiveness to FSH therapy (when available), are reported in **Table 2**. Unpredictably, after genetic testing and a more than a 2 year-long history of couple infertility, patients 5 (despite mild oligozoospermia) and 8 (despite oligozoospermia and testicular hypotrophy) spontaneously impregnated their wives, fathering healthy children.

**Table 2:** Clinical features of the patients positive for pre-diagnostic genes.

	Gene(s)	Clinical suspect	Sperm parameters^1^	FSH serum levels (IU/ml)	Testicular volume (right and left)^2^	Testicular histology	FSH responsiveness^3^
**Subject 1**	*DNAH11, DNAI1, GALNTL5*	Primary defects of spermatogenesis	Mild OAT	6.6	9.6 ml and 14.9 ml	—	No
**Subject 2**	*DNAH5, AMELY*	Primary defects of spermatogenesis	Azoospermia	8.0	19.5 ml and 19.9 ml	NA	NA
**Subject 3**	*CCDC40*	Primary defects of spermatogenesis	OAT	5.4	9.8 ml and 11.2 ml	—	NA
**Subject 4**	*DNAH10*	Primary defects of spermatogenesis	?	?	?	?	?
**Subject 5**	*KLK4*	Primary defects of spermatogenesis	Mild OAT	3.6	15.1 ml and 11.7 ml	—	NA
**Subject 6**	*DNAH10*	Primary defects of spermatogenesis	Mild OAT	7.3	7.5 ml and 12.6 ml	—	No
Subject 7^*^	*DNAH11*	Primary defects of spermatogenesis	Normozoospermia	5.7	10.9 ml and 10.7 ml	—	Yes
**Subject 8**	*CFTR*	Primary defects of spermatogenesis	OAT	16.3	6.3 ml and 9.8 ml	—	—
**Subject 9**	*CATSPER2, KLK14*	Primary defects of spermatogenesis	Azoospermia	32.7	6.7 ml and 8.7 ml	Sertoli cell only syndrome	—
**Subject 10**	*ADCY10*	Primary defects of spermatogenesis	OAT	3.6	10.1 ml and 12.5 ml	—	No

^1^Assessed using WHO 2010 guidelines.

^2^Evaluated by ultrasound (ml).

^3^FSH responsiveness was defined by the doubling of sperm concentration or total sperm count vs. pre-treatment values.

*The patient was diagnosed for reversal central hypogonadism. The values shown have been measured following 5 months from treatment withdrawal.

Severe oligozoospermia was defined for total sperm count <1.0 million; mild oligozoospermia was defined for total sperm count enclosed between 1.0 and 5.0 million; oligozoospermia for total sperm count enclosed between 5.0 and 39.0 million (21).

FSH, follicle-stimulating hormone; NA, not available; OAT, oligo-astheno-teratozoospermia.

Our gene panel design generated a mean sequencing depth of 359X, whereas 98% of the target regions had a sequencing depth of at least 25X. Variants in the pre-diagnostic genes were identified in 10/12 subjects negative to diagnostic testing with suspected defects of primary spermatogenesis (83%). Seventeen filtered variants were detected in 12 of the 65 genes analyzed (18%): *DNAH11*, *DNAH10*, *DNAH5*, *DNAI1*, *CCDC40*, *CFTR*, *GALNTL5*, *AMELY*, *KLK4*, *KLK14*, *CATSPER2*, and *ADCY10*. In particular, two heterozygous variants (p.Lys1853*, rs748618094, in *DNAH5* and p.Asp1152His, rs75541969, in *CFTR*) already reported as pathogenic were detected. Three variants with uncertain significance: p.Arg654Cys, rs140820295 in *DNAI1* (heterozygous); p.Pro3935Leu, rs72658814 in *DNAH11* (homozygous); and p.Asp284His, rs201042940 in *CCDC40* (heterozygous) were also found. All of them were predicted to be disease-causing by MutationTaster, Damaging by SIFT, and Probably Damaging by Polyphen-2.

Moreover, three variants with high impact were identified: the hemizygous splice variant c.574-1G>A (rs760519968) in *AMELY* affects the acceptor splice site of the last exon and may cause the activation of a cryptic splice site and consequently a stop-loss mutation. This variant is predicted to be disease-causing by MutationTaster. The heterozygous variant c.842+1G>C (rs199516208) in *CATSPER2* affects a donor splice site. This may cause the activation of a cryptic splice site and the introduction of a premature stop codon and is considered disease-causing by MutationTaster. The heterozygous truncating variant c.90T>A; p.Cys30* in *ADCY10*. This variant is considered pathogenic for the autosomal dominant inherited condition of susceptibility to absorptive hypercalciuria (OMIM #143870).

**Figure 2:**
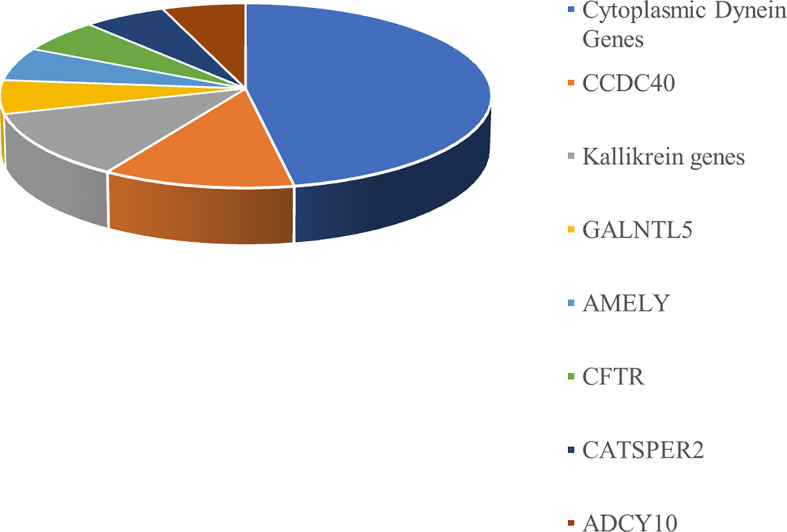
Pre-diagnostic gene variants distribution.

The genetic variants identified in the 12 infertile patients enrolled in this study using an NGS pre-diagnostic genes panel are reported in **Table 3**. Almost half of the variants identified by NGS in the 12 patients included in this study belong to the cytoplasmic dynein genes. The distribution of pre-diagnostic genes variants is shown in **Figure 2**.

**Table 3:** Genetic variants of the pre-diagnostic genes identified in infertile patients negative to an NGS diagnostic test consisting of 110 genes.

	Gene	HGVS^1^ cDNA	HGVS^1^ protein	Reference ID according to NCBI	Consequence	Clinic relevance^2^	*In silico* prediction	ClinVar accession
**Subject 1**	*DNAH11*	NM_001277115.1:c.5805G>C	NP_001264044.1:p.Leu1935Phe	–	missense variant	–	deleterious	SCV001432675
	*DNAI1*	NM_001281428.1:c.1960C>T	NP_001268357.1:p.Arg654Cys	rs140820295	missense variant	uncertain significance	deleterious	SCV001432676
	*GALNTL5*	NM_145292.3:c.1256G>C	NP_660335.2:p.Arg419Pro	–	missense variant	–	deleterious	SCV001432677
**Subject 2**	*DNAH5*	NM_001369.2:c.5557A>T	NP_001360.1:p.Lys1853Ter	rs748618094	stop gained	pathogenic	–	SCV001432678
	*AMELY*	NM_001143.1:c.574-1G>A	–	rs760519968	splice acceptor variant	–	–	SCV001432679
**Subject 3**	*CCDC40*	NM_001243342.1:c.1945T>C	NP_001230271.1:p.Phe649Leu	–	missense variant	–	deleterious	SCV001432680
	*CCDC40*	NM_001243342.1:c.850G>C	NP_001230271.1:p.Asp284His	rs201042940	missense variant	uncertain significance	deleterious	SCV001432681
**Subject 4**	*DNAH10*	NM_207437.3:c.10174C>G	NP_997320.2:p.Pro3392Ala	rs143987578	missense variant	–	deleterious	SCV001432682
**Subject 5**	*KLK4*	NM_001302961.1:c.395C>T	NP_001289890.1:p.Pro132Leu	rs144350395	missense variant	–	deleterious	SCV001432683
**Subject 6**	*DNAH10*	NM_207437.3:c.10954G>A	NP_997320.2:p.Ala3652Thr	–	missense variant	–	deleterious	SCV001432684
	*DNAH10*	NM_207437.3:c.3514C>T	NP_997320.2:p.Leu1172Phe	rs778218750	missense variant	–	deleterious	SCV001432685
	*DNAH10*	NM_207437.3:c.3221A>G	NP_997320.2:p.Asn1074Ser	rs771006247	missense variant	–	benign	SCV001432686
**Subject 7**	*DNAH11*	NM_001277115.1:c.11804C>T	NP_001264044.1:p.Pro3935Leu	rs72658814	missense variant	uncertain significance	deleterious	SCV001432687
**Subject 8**	*CFTR*	NM_000492.3:c.3454G>C	NP_000483.3:p.Asp1152His	rs75541969	missense variant	pathogenic & drug response	deleterious	SCV001432688
**Subject 9**	*CATSPER2*	NM_001282309.2:c.842+1G>C	–	rs199516208	splice donor variant	–	–	SCV001432689
	*KLK14*	NM_001311182.1:c.700G>A	NP_001298111.1:p.Val234Met	rs201317571	missense variant	–	deleterious	SCV001432690
**Subject 10**	*ADCY10*	NM_001297772.1:c.90T>A	NP_001284701.1:p.Cys30Ter	–	stop gained	–	–	SCV001432691

^1^All identiﬁed variants are indicated both by cDNA base sequence (third column) and by protein sequence (fourth column) according to the HGVS (Human Genome Variation Society) nomenclature guidelines.

^2^Information reported in NCBI (National Centre for Biotechnology Information) database.

## Discussion

Male infertility is a condition with highly heterogeneous phenotypic representation and a complex multifactorial etiology including environmental and genetic factors. The elevated number of candidate genes makes it hard to find a genetic cause of infertility in the majority of the cases (22–24). Anyway, a multi-disease gene panel can improve the identification of the etiology of male infertility (3, 25, 26). In several cases, idiopathic infertility has a genetic origin, therefore a correct phenotyping and medical history of the infertile patient may represent an initial basis for the genetic interpretation of the disorder (27), especially for the genetic variants of uncertain significance (VUS). To classify genetic variants, a prior likelihood of pathogenicity, based on *in silico* analysis, can be associated with the available genetic and epidemiological data to calculate the probability that a variant is pathogenic, in a multifactorial likelihood model.

Based on references of the American College of Medical Genetics and Genomics, genetic variants can be distinguished into five classes: pathogenic, likely pathogenic, variant of uncertain significance, likely benign, or benign (28). A VUS is a genetic change with unclear implications for gene function. Interpretation of VUS represents a difficult challenge for genetic counseling and clinical management of infertile male patients. It is fundamental to identify VUS and to evaluate them since, at moment, they are not clearly associated with a phenotype but may be classified as pathogenic in the future (29–31).

We have successfully developed a genetic test based on NGS that covers the main male infertility indications (9, 32, 33). We developed a custom-made panel of 65 additional pre-diagnostic genes that we tested in 12 infertile patients who were negative to a diagnostic panel consisting of 110 genes. Eleven patients had a primary spermatogenic failure and one patient had central hypogonadism.

In our analysis, 17 filtered variants were found in the following 12 out of the 65 genes analyzed (18%): *DNAH11*, *DNAH10*, *DNAH5*, DNAI1, *CCDC40*, *CFTR*, *GALNTL5*, *AMELY*, *KLK4*, *KLK14*, *CATSPER2*, and *ADCY10*. Some reports have described the involvement of the mutations of these genes in the pathogenesis of male infertility. As an example, *DNAH11*, *DNAH5*, *DNAI1*, and *CCDC40* genes have been linked to primary ciliary dyskinesia (34, 35). Similarly, the *GALNTL5* and the *KLK* genes may be involved in the pathogenesis of asthenozoospermia (36, 37).

Almost half of the variants identified by NGS belong to the cytoplasmic dynein genes (**Figure 2**). Dynein genes are known to be involved in the syndromic forms of asthenozoospermia, including primary ciliary dyskinesia/Kartagener syndrome (38–40). A possible association between variants of dynein genes and isolated non-syndromic asthenozoospermia has also been reported (41).

Two pathogenic variants in two patients with primary spermatogenic failure were identified: p.Lys1853*, rs748618094 in *DNAH5*, and p.Asp1152His, rs75541969 in *CFTR* (42). *DNAH5 (*Dynein Axonemal Heavy Chain 5), mapping on the chromosome 5p15.2, encodes an axonemal heavy chain dynein protein. Variations in this gene mainly cause primary ciliary dyskinesia type 3 and Kartagener syndrome, which are diseases due to ciliary defects. Truncating variants in *DNAH5* results in the absence of the outer dynein arm of the cilia, leading to abnormal ciliary structure and motor function (43, 44). In this specific case, Subject 2 has azoospermia and carries this variant in a heterozygous state, a trait that may be associated with mutations in *DNAH5*. However, pathologic phenotype associated with mutations in *DNAH5* is inherited in a recessive manner. We cannot exclude the presence of a large deletion/insertion in the other allele or the contribution of other genes. *CFTR* (CF Transmembrane Conductance Regulator), mapping on chromosome 7q31.2, encodes a membrane protein and chloride channel. Notoriously, mutations in this gene cause cystic fibrosis (45). *CFTR* is important for spermatogenesis (46). Genetic variants of the *CFTR* gene are a relatively frequent cause of male infertility, due to obstructive azoospermia, or in atypical forms of CF such as the congenital absence of the vas deferens, bilateral ejaculatory duct obstruction, or bilateral obstructions (47, 48). However, the patient studied here (Subject 8) has oligo-astheno-teratozoospermia, a trait never associated with this gene. We cannot exclude the presence of a large deletion/insertion in the other allele or the contribution of other genes.

Moreover, in our analysis three VUS were found: p.Arg654Cys, rs140820295 in *DNAI1*, p.Pro3935Leu, rs72658814 in *DNAH11*, and p.Asp284His, rs201042940 in *CCDC40*.

*DNAI1* (Dynein Axonemal Intermediate Chain 1), mapping on the chromosome 9p13.3, and *DNAH11* (Dynein Axonemal Heavy Chain 11), mapping on the chromosome 7p15.3, are other genes of the dynein family related to primary ciliary dyskinesia and involved in male infertility (48), especially in isolated non-syndromic asthenozoospermia (32). The variant in *DNAI1* is heterozygous; however primary ciliary dyskinesia caused by mutations in *DNAI1* is inherited in an autosomal recessive manner. We cannot exclude that heterozygous variants in *DNAI1* may cause a milder phenotype characterized only by infertility. In this specific case, Subject 1 showed oligo-astheno-teratozoospermia. Variants of *DNAH11* are found also in primary ciliary dyskinesia patients with normal ciliary ultrastructure. Interestingly, we found a patient (Subject 7) that carries the p.Pro3935Leu variant in a homozygous state. In gnomAD this variant is always reported in a heterozygous state. *CCDC40* (Coiled-Coil Domain Containing 40) mapping on the chromosome 17q25.3, is another gene associated with ciliary dyskinesia. The coiled-coil domain-containing protein CCDC40 is essential for motile cilia function and left-right axis formation (49). The variant p.Asp284His was found in compound heterozygosity with p.Phe649Leu, therefore we may speculate that both variants cannot cause major developmental defects like primary ciliary dyskinesia but they can cause oligo-astheno-teratozoospermia as observed in Subject 3. Interestingly, other variants with high impact requiring further functional and family segregation studies were identified. For instance, the splice variants rs760519968 in *AMELY* and rs199516208 in *CATSPER2*, and the stop gained variant p.Cys30* in *ADCY10.* To date, no loss-of-function mutations have been reported in the *AMELY* (Amelogenin Y-linked) gene in association with infertility. Structural rearrangements involving *AMELY*, mapping on the chromosome Yp11.2, have been found in patients with hypogonadism (50), although a direct link between the phenotype and the rearrangement has not been proven. *CATSPER2* (Cation Channel Sperm Associated 2) mapping on the chromosome 15q15.3 is the main Ca^2+^ channel mediating extracellular Ca^2+^ influx into spermatozoa. *CATSPER*-related infertility is associated with azoospermia. This is consistent with the phenotype reported in Subject 9 (51). *ADCY10* (Adenylate Cyclase 10) mapping on the chromosome 1q24.2, encodes for soluble adenylyl cyclase, which is the predominant adenylate cyclase in sperm crucial to sperm motility regulation, and it is associated with severe recessive asthenozoospermia (52). Subject 10 shows oligo-astheno-teratozoospermia, therefore his phenotype is partially overlapping with asthenozoospermia. Although truncating variants in *ADCY10* are recessively inherited when associated with infertility, we cannot exclude the presence of a large insertion/deletion in the other allele that was not detected with NGS.

Therefore, an NGS custom-made panel test including pre-diagnostic genes can give an improvement to genetic diagnostic testing and can influence male infertility clinical management. The precise prevalence of male infertility is not known and, at present, there are not complete systematic reviews or meta-analyses on the epidemiology of male infertility (53, 54). Making the diagnosis of genetic infertility is of relevance, also because the available epidemiological observations indicate lower life expectancy and higher morbidity in infertile patients (55, 56).

In conclusion, we showed the efficacy of NGS-based approaches also employing pre-diagnostic genes. This panel of genes may help to identify the etiology underlying the disorder and guide clinical management.

## Data Availability Statement

The dataset presented in this study can be found in online repositories. The names of the repository/repositories and accession numbers can be found in the article/supplementary material.

## Ethics Statement

The experimental protocol was performed in the Division of Andrology and Endocrinology of the Teaching hospital “G. Rodolico,” University of Catania, Catania, Italy. The internal Institutional Review Board approved the study protocol. An exhaustive explanation of the study purpose was given to each participant and informed written consent was obtained in compliance with Helsinki’s declaration. The patients/participants provided their written informed consent to participate in this study.

## Author Contributions

VP wrote the article. RC collected clinical data and critically revised the article. SP, GMB, TB, LS, GT, and AZ analyzed the data and critically revised the article. GM performed the bioinformatic analysis and critically revised the article. AEC conceived the study, collected clinical data, supervised the work, and critically revised the article. MB conceived the study, supervised the work, and critically revised the article. All authors contributed to the article and approved the submitted version.

## Funding

This work was supported by funding from the Provincia Autonoma di Trento within the initiative LP6/99 (dpg 1045/2017).

## Conflict of Interest

Authors SP, AZ, and MB were employed by the company MAGI’S LAB.

The remaining authors declare that the research was conducted in the absence of any commercial or financial relationships that could be construed as a potential conflict of interest.
